# Use of a high-density mapping catheter for Purkinje-related ventricular tachycardia in a patient with a previous history of anterior myocardial infarction

**DOI:** 10.1016/j.hrcr.2021.01.007

**Published:** 2021-01-20

**Authors:** Yousaku Okubo, Yukimi Uotani, Shogo Miyamoto, Shunsuke Miyauchi, Yoshihiro Ikeuchi, Sho Okamura, Takehito Tokuyama, Yukiko Nakano

**Affiliations:** Department of Cardiovascular Medicine, Hiroshima University Graduate School of Biomedical and Health Sciences, Hiroshima, Japan

**Keywords:** Catheter ablation, High-density 3D mapping, Mid-diastolic potential, Purkinje-related ventricular tachycardia, Ventricular tachycardia after myocardial infarction

## Introduction

Monomorphic ventricular tachycardia (VT) in patients with a previous history of a myocardial infarction (MI) is commonly caused by reentry circuits in myocardial scar area or its borders. However, in some cases, VTs after MI have reentrant circuits mediated by Purkinje fibers: bundle branch reentry, interfascicular reentry, intrafascicular reentry, and focal Purkinje VT.^1^ Bogun and colleagues^2^ assessed 81 consecutive patients with monomorphic VT after MI. They found that Purkinje fibers may play a major role in the reentry circuit of postinfarction VT characterized by narrow QRS morphologies. Moreover, Hayashi and colleagues^3^ investigated the monomorphic VTs after MI, named left posterior Purkinje reentry, which were similar to verapamil-sensitive VT, and found that VTs were successfully ablated at the site with presystolic or diastolic Purkinje potentials. Although the aforementioned Purkinje potential plays a critical role in the VT circuit, the entire reentrant mechanism of the left posterior Purkinje reentry is still unclear.^3^ Herein, we report a case of Purkinje fiber–mediated VT after an MI that was successfully treated with catheter ablation using a high-density (HD) mapping catheter (Advisor™ HD Grid Mapping Catheter; Abbott, St. Paul, MN).

**Key Teaching Points**

• Purkinje-related ventricular tachycardia (VT) after myocardial infarction (MI) is similar to left posterior fascicular VT, as the reentry circuit includes the Purkinje fiber network.
• Catheter ablation targets the mid-to-late diastolic potential (P1), which is a sharp and high-frequency potential preceding the Purkinje potential (P2). However, the P1 potentials are only observed in less than one-third of all cases. Moreover, these potentials and critical isthmus during VT can be recorded using the Adviser HD Grid Mapping Catheter (Abbott, St. Paul, MN), but not with the conventional linear duodecapolar catheter.
• The HD Grid catheter is useful for mapping not only scar-related VT after MI but also Purkinje-related VT, which requires a precise mapping of the Purkinje fiber network.

## Case report

**Figure 1:**
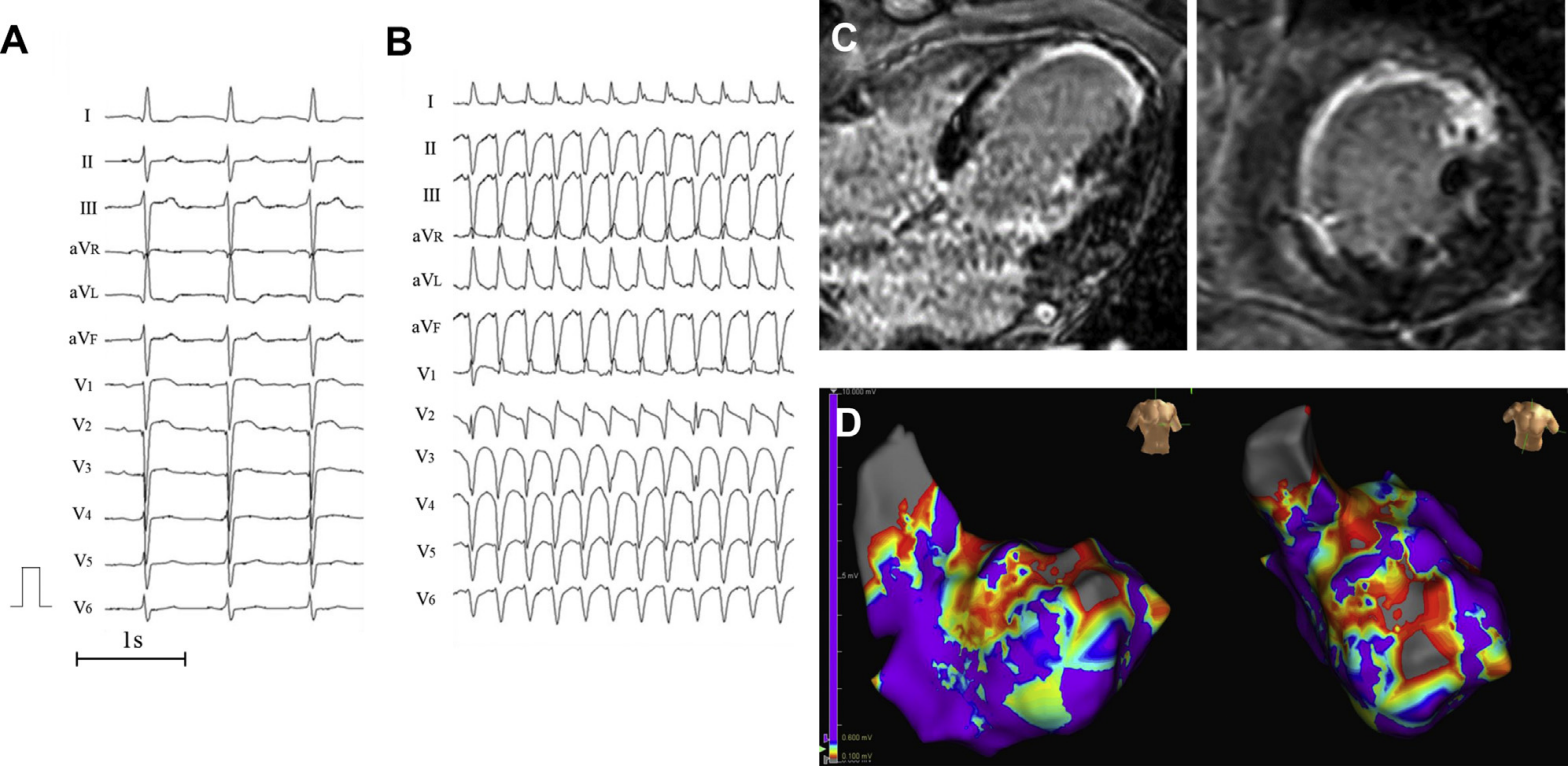
**A:** Findings of the 12-lead electrocardiogram during normal sinus rhythm. QRS complexes during sinus rhythm revealed the presence of a left anterior fascicular block. **B:** Results of the 12-lead electrocardiogram during ventricular tachycardia: fast heart rate (200 beats per minute), narrow QRS interval (121 ms), right bundle branch block, and left axis deviation. **C:** Magnetic resonance imaging revealed a broad transmural scar in the septum and anterior wall of the left ventricle owing to a previous myocardial infarction. **D:** Endocardial left ventricle voltage mapping during sinus rhythm showed a wide area with low voltages around the infarct scars. Low-voltage area (*red*) and dense scar area (*gray*) were defined as a peak-to-peak bipolar voltage of 0.1–0.6 and <0.1 mV, respectively.

A 69-year-old man was transferred to our hospital owing to a tachycardia causing a hemodynamic collapse, which was successfully terminated by electrical cardioversion. The patient had a history of an extensive anterior MI diagnosed 20 years back. However, he did not undergo revascularization procedure. The electrocardiogram conducted before the cardioversion showed a sustained VT, fast heart rate (200 beats per minute), narrow QRS interval (121 ms), right bundle branch block, and left axis deviation (Figure 1B). The QRS polarities during sinus rhythm were similar to those during the VT (Figure 1A).

Cardiac echocardiography and magnetic resonance imaging revealed a large transmural scar on the septum and anterior wall of the left ventricle (LV) and reduced left ventricular ejection fraction (ejection fraction = 37%) owing to a prior MI (Figure 1C). During hospitalization, the patient had several episodes of sustained monomorphic VT. Several studies have reported that catheter ablation is the most effective first-line therapy for scar-related VT.^4^ Hence, in the current case, this procedure was performed.

In the electrophysiological examination, we placed a 5F decapolar electrode catheter in the coronary sinus via the right internal jugular vein (Inquiry™ Luma-Cath™; Abbott, St. Paul, MN) and at the His bundle site (Supreme™; Abbott, St. Paul, MN) and a duodecapolar electrode catheter (Inquiry Livewire™; Abbott, St. Paul, MN) in the LV via the right femoral artery. After a routine electrophysiological study, endocardial left ventricular voltage mapping during sinus rhythm was performed using the Livewire and EnSite™ NavX™ systems (Abbott, St. Paul, MN). The low-voltage area and dense scar area were defined as peak-to-peak bipolar voltages of 0.1–0.6 and <0.1 mV, respectively. The voltage map revealed a wide area with low voltages around the infarct scars (Figure 1D). After the creation of the voltage map, a VT, which was an exact match of the clinical VT, was induced by rapid ventricular burst pacing from the right ventricular apex. During the VT, atrioventricular dissociation was observed, and the VT was hemodynamically unstable (cycle length of 290 ms). The HV intervals during sinus rhythm and the VT were 46 and −40 ms, respectively. We performed entrainment pacing from the right ventricular apex at a cycle length that was 20 ms shorter than the VT cycle length. The morphology of paced QRS showed constant fusion, with a constant waveform between the complete paced QRS and VT QRS, suggesting that the mechanism of the VT was reentrant. The postpacing interval after entrainment pacing was 50 ms greater than the tachycardia cycle length of VT.

**Figure 2:**
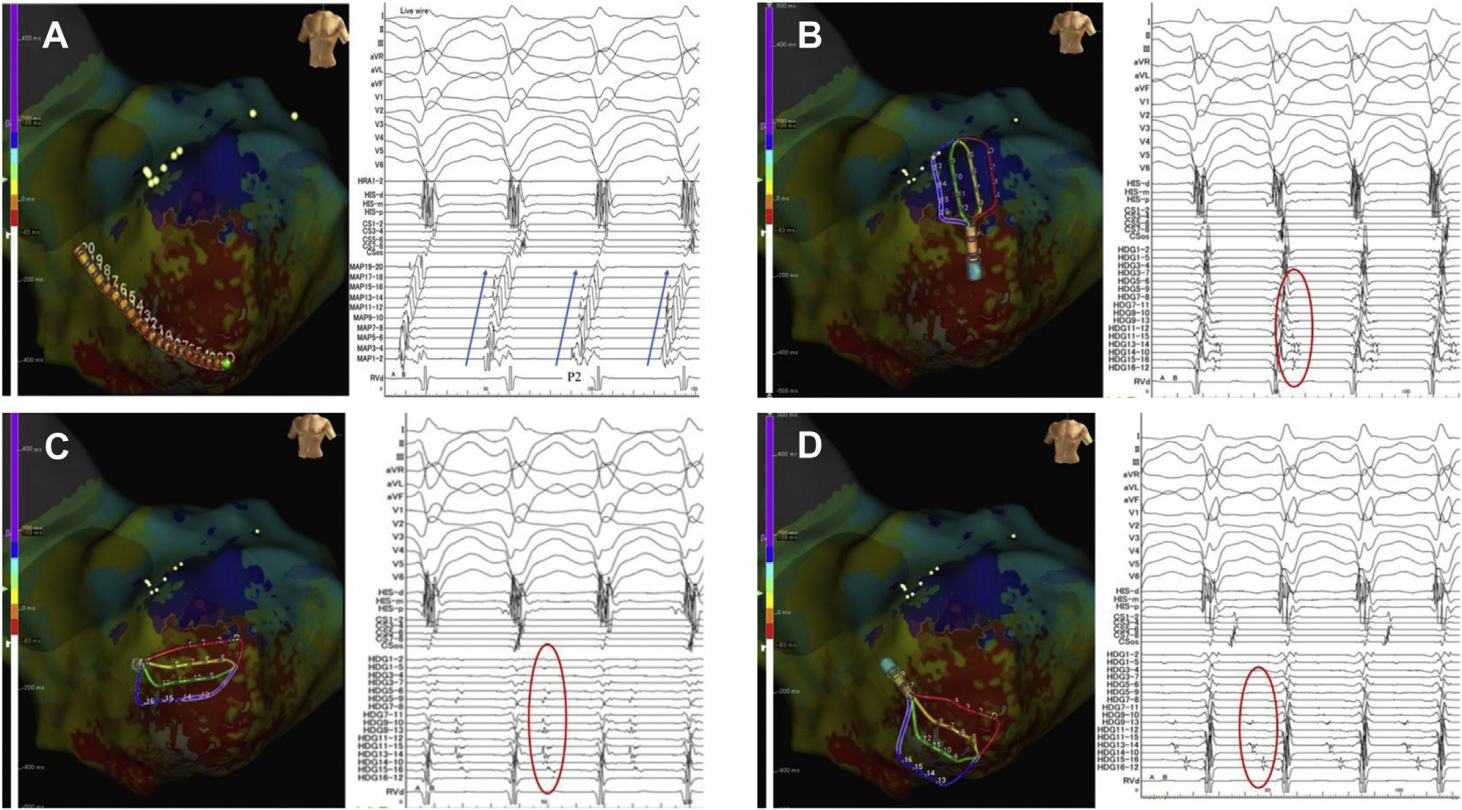
**A:** A duodecapolar electrode catheter (Inquiry Livewire; Abbott, St. Paul, MN) located at the apical-inferior septum of the left ventricle could record left posterior fascicular potentials during sinus rhythm. Moreover, it revealed a presystolic potential (P2) during ventricular tachycardia. The P2 potentials exhibited retrograde conduction via the left posterior Purkinje fiber (*blue arrow*). **B:** The Advisor HD Grid Mapping Catheter (Abbott, St. Paul, MN) located on the anterior wall of the left ventricle exhibited late potentials during the ventricular tachycardia (*red circle*). **C:** The Advisor HD Grid Catheter located on the left ventricular apical-anterior wall exhibited the mid-diastolic potentials during ventricular tachycardia (*red circle*). Some potentials were small and had dull signals representing the far-field signals of the epicardium. **D:** The Advisor HD Grid Mapping Catheter located at the apical-inferior septum of the left ventricle exhibited at the mid-to-late diastolic potentials, which could not be recorded with a duodecapolar electrode catheter.

The duodecapolar electrode catheter (Inquiry Livewire, Abbott, St. Paul, MN) located at the apical-inferior septum of the LV could record left posterior fascicular potentials during sinus rhythm. It showed a presystolic potential (P2) during the VT (Figure 2A). We attempted entrainment pacing at that location, but it was not achieved because the VT accelerated and required multiple cardioversions. Thus, we performed pace mapping during sinus rhythm. We found that the stimulus QRS interval was similar to the EGM-QRS interval and the QRS morphology during pace mapping was similar to that of the VT. Since entrainment pacing was not possible, we could not prove that this location was the exit of the VT reentry circuit, but the results of the activation mapping and pace mapping seem to support the possibility of this location being the exit of the VT circuit.

**Figure 3:**
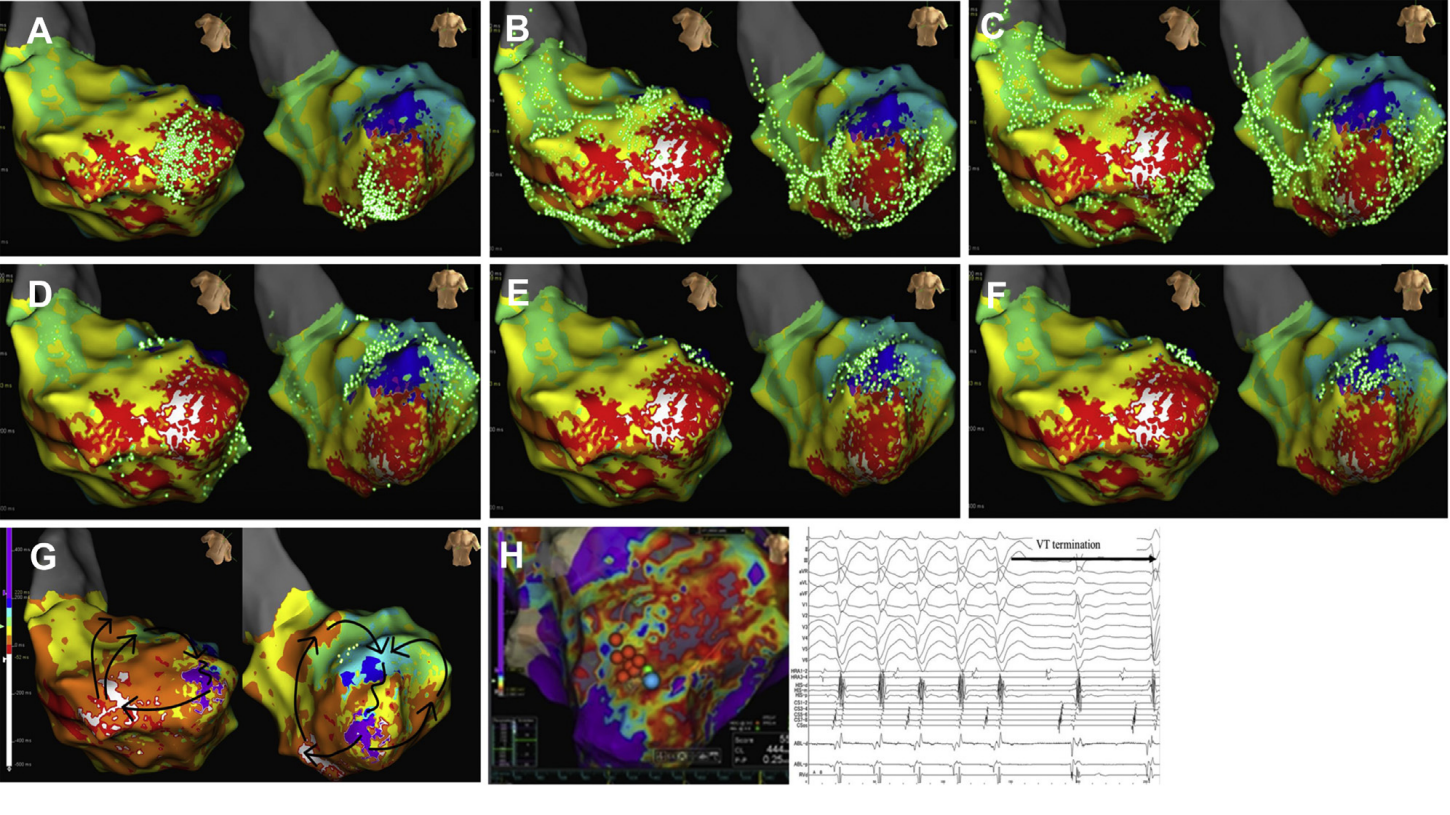
Activation pattern of ventricular tachycardia (VT). **A–G:** The activation map revealed that the impulse propagated retrogradely from the VT exit site to the left posterior Purkinje fibers and another propagated from the VT exit site to the apical-anterior wall site via the endocardium. The impulse that propagated retrogradely via the Purkinje fiber entered from the endocardial side of the apical-anterior wall region surrounded by the scar regions, then propagated on the epicardium to the septum, and finally exited from a distal site of the left posterior fascicule area. **H:** Successful ablation site of the VT. The radiofrequency energy (30 W) was applied to the VT isthmus site using an 8F, 4-mm flexible irrigated-tip catheter with 1-4-1-mm interelectrode spacing (FlexAbility Ablation Catheter, Abbott, St. Paul, MN). The VT terminated and could no longer be induced by an isoproterenol infusion or programmed stimulation.

The VT was hemodynamically unstable and the patient’s systolic blood pressure dropped below 60 mm Hg. Instead of using mechanical circulatory support, the depth of sedation was modified from deep sedation to moderate (conscious) sedation and intravenous fluid bolus was used as needed to maintain the mean arterial pressure > 60 mm Hg. Thus, we continued activation mapping during the VT. Although we cautiously mapped the low-voltage area using the linear duodecapolar catheter during the VT, no mid-to-late diastolic potentials (P1) could be recorded (Supplemental Figures 1 and 2). Thus, we performed local activation time (LAT) mapping using an HD mapping catheter (Advisor HD Grid Mapping Catheter). The earliest LAT point was −54 ms, and the latest LAT point was 228 ms, thereby covering the entire tachycardia cycle length. We could reproducibly record the mid-to-late diastolic potentials in the apical-anterior wall of the LV during VT (Figure 2B and 2C) Moreover, we could record P1 potentials using the HD grid catheter (Figure 2D). During the pre–systolic phase, the impulse propagated retrogradely from the VT exit site to the left posterior Purkinje fibers, and simultaneously another propagated from the VT exit site to the apical-anterior wall site via the endocardium (Supplemental Figure 3A and 3B). Then, during the mid-to-late diastolic phase, the impulse propagated from the anterior wall to the septal wall site (Supplemental Figure 4A and 4B). In this area, the impulse propagated in the opposite direction (Supplemental Figure 5) and some potentials during the mid-to-late diastolic phase were small, dull signals representing the far-field signals of the epicardium. Although we did not perform activation mapping in the epicardium of the LV and simultaneous mapping of the right ventricle, the aforementioned findings suggested that the VT circuit propagated on the epicardium of the anterior wall. The detailed activation map revealed that the impulse entered from the endocardial side of the apical-anterior wall region surrounded by scar regions, then propagated on the epicardium to the septum, and finally exited from the distal site of the left posterior fascicule area (Figure 3A–3G, Supplemental Video File). Then, we positioned an 8F, 4-mm flexible irrigated-tip catheter with 1-4-1-mm interelectrode spacing (FlexAbility™ Ablation Catheter, Abbott, St. Paul, MN) on the VT entrance. While applying a radiofrequency energy application (30 W) from the site of the VT entrance to the VT exit including the VT isthmus, the VT terminated and could no further be induced by an isoproterenol infusion or programmed stimulation (Figure 3H). After the procedure, the patient did not experience any VT episodes during the 12-month follow-up period, without the administration of antiarrhythmic agents.

## Discussion

Herein, we report a case of Purkinje-related VT after an MI that was successfully treated with catheter ablation. Recently, several studies have shown that VTs with a relatively narrow QRS duration, which have characteristics similar to those of left posterior fascicular VT, occur after an MI.^2^^,^^3^

Catheter ablation targets the P1 potential, a sharp and high-frequency potential preceding the P2 potential. However, the proportion of patients with P1 potentials is not extremely high.^5^ Liu and colleagues^5^ showed that the P1 fiber is located near or parallel to the left posterior Purkinje fiber in most patients with P1 potentials. Therefore, a P1 cannot be recorded, probably owing to both technical issues with the mapping and anatomical problems based on the changes in the length and orientation of the P1 fiber. Moreover, linear catheters may have poor contact when recording the P1 potentials owing to complex structures including endocardial trabeculations. The novel mapping catheter (Advisor HD Grid) is composed of 16 equidistant electrodes arranged in a 4 × 4 grid, allowing high-density bipolar recordings along and across the splines in a short period. Proietti and colleagues^6^ showed that this novel mapping catheter can obtain more efficient and detailed data about the critical isthmus site within the low-voltage area than the conventional linear bipolar catheter. Since the bipolar amplitudes are dependent on the angle of the wavefront, the amplitude of the electrocardiograms recorded by an ablation catheter or linear duodecapolar catheter might be pseudo-low voltage. Jiang and colleagues^7^ demonstrated that the local amplitudes recorded by the HD grid catheter are higher than that recorded by a conventional linear mapping catheter. In the current case, using an HD grid catheter, we could record the P1 potentials, create a detailed VT activation map, and reveal the critical isthmus site within the low-voltage area. Furthermore, the activation map of the VT revealed retrograde conduction via the left posterior Purkinje fiber that propagated from the septal wall to the anterior wall, entered to the slow conduction area surrounded by the low-voltage area, and propagated to the distal site of the Purkinje fiber again. Purkinje-related VTs in patients with a post-MI are similar to left posterior fascicular VTs in which the reentry circuit includes the Purkinje fiber network. However, similar to other scar-related VTs, the critical isthmus site was located near the scar border. During the application of radiofrequency energy to the critical isthmus, the VT was terminated. In the current case involving left anterior fascicular block, owing to the ablation site being away from the left posterior fascicular region, progression to complete left bundle branch block was prevented. To the best of our knowledge, this is the first case report on a precise activation map of a Purkinje-related VT after an MI using an HD grid mapping catheter.

## Conclusion

An HD grid catheter is useful for mapping not only MI scar–related VTs but also Purkinje-related VTs, which requires a precise mapping of the Purkinje fiber network.

## Appendix Supplementary data

Supplementary Figure Legend

Supplemental Video File Activation pattern of ventricular tachycardia (VT). The activation map revealed that the impulse propagated retrogradely from the VT exit site to the left posterior Purkinje fibers and another propagated from the VT exit site to the apical-anterior wall site via the endocardium. The impulse that propagated retrogradely via the Purkinje fiber entered from the endocardial side of the apical-anterior wall region surrounded by the scar regions, then propagated on the epicardium to the septum, and finally exited from a distal site of the left posterior fascicule area.
