# Supplementary material for: Use of a high-density mapping catheter for Purkinje-related ventricular tachycardia in a patient with a previous history of anterior myocardial infarction
Source: HeartRhythm Case Rep. 2021 Jan 20;7(4):232–6. doi: 10.1016/j.hrcr.2021.01.007 (PMC8129040; doi:10.1016/j.hrcr.2021.01.007)
Supplement: Supplemental Figures [file mmc1.pdf]

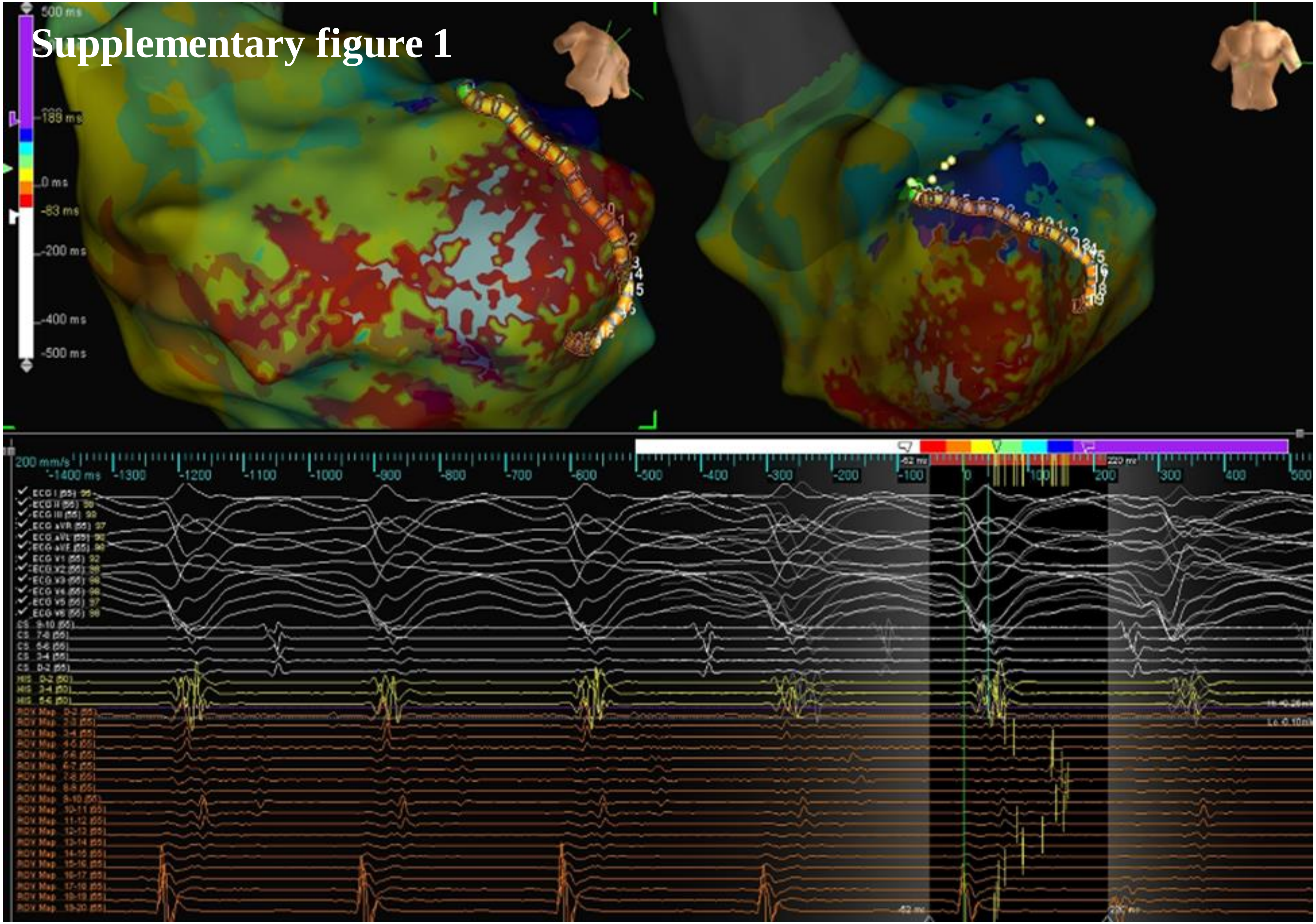

Supplementary figure 1

Supplementary figure 2

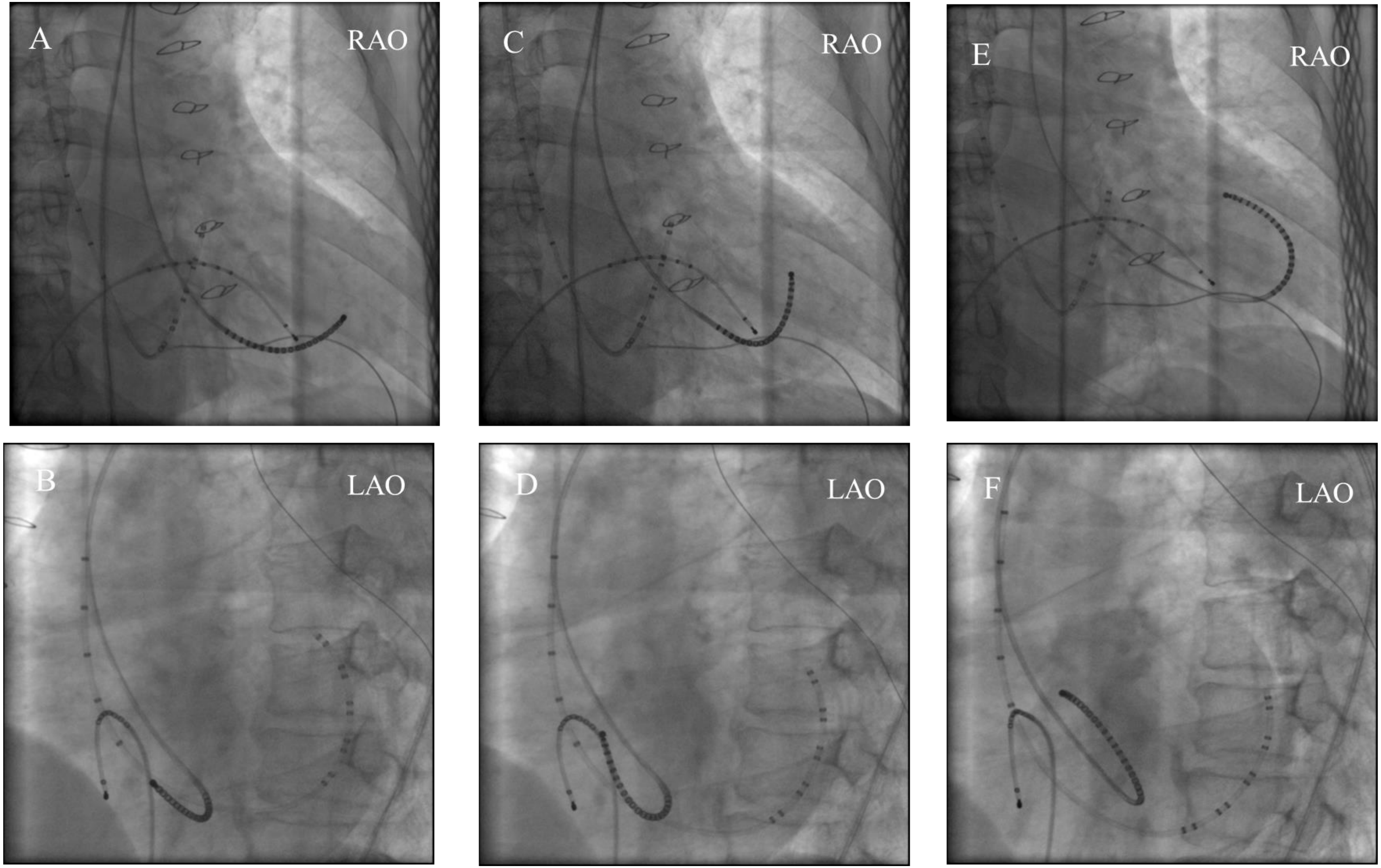

Supplementary figure 3

A

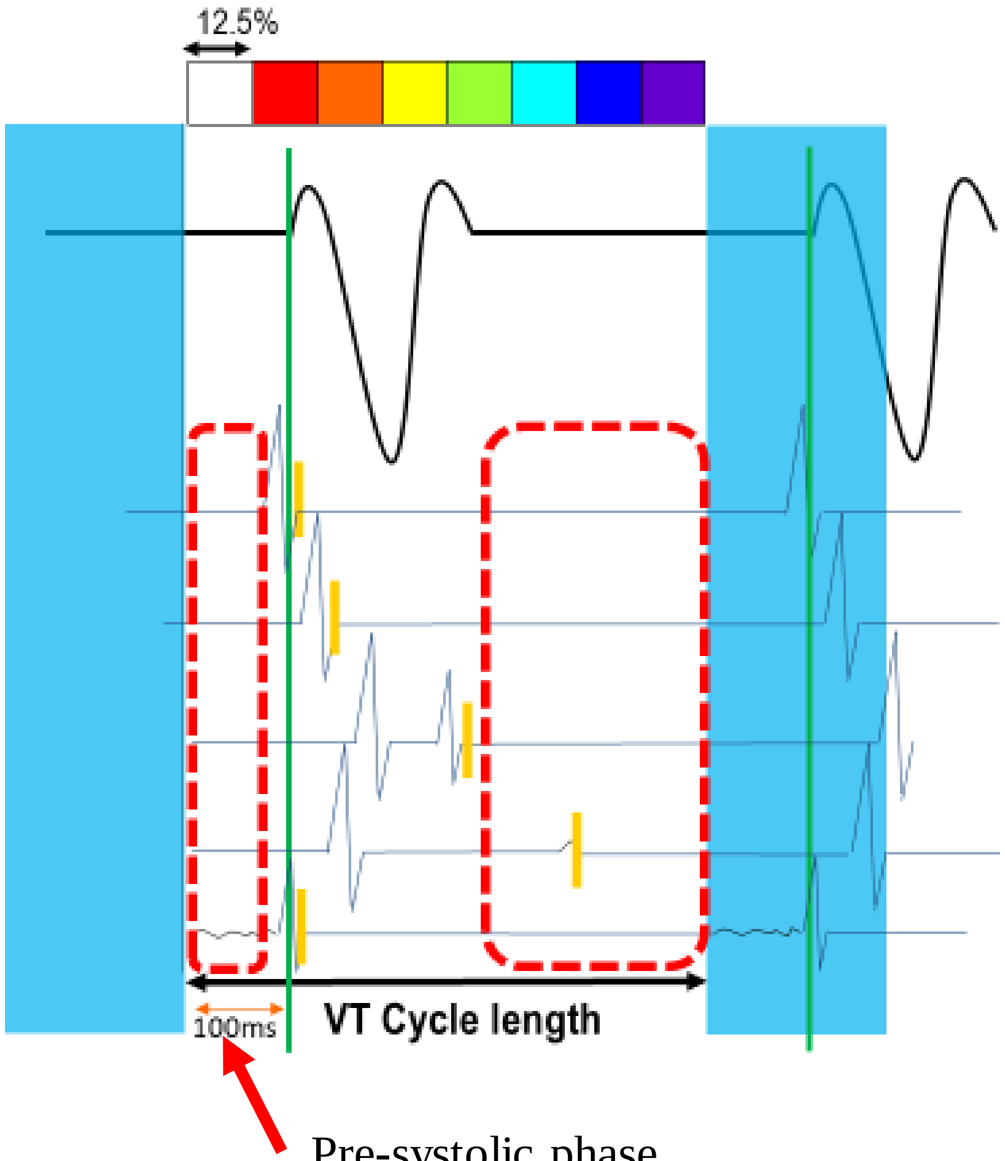

Pre-systolic phase

B

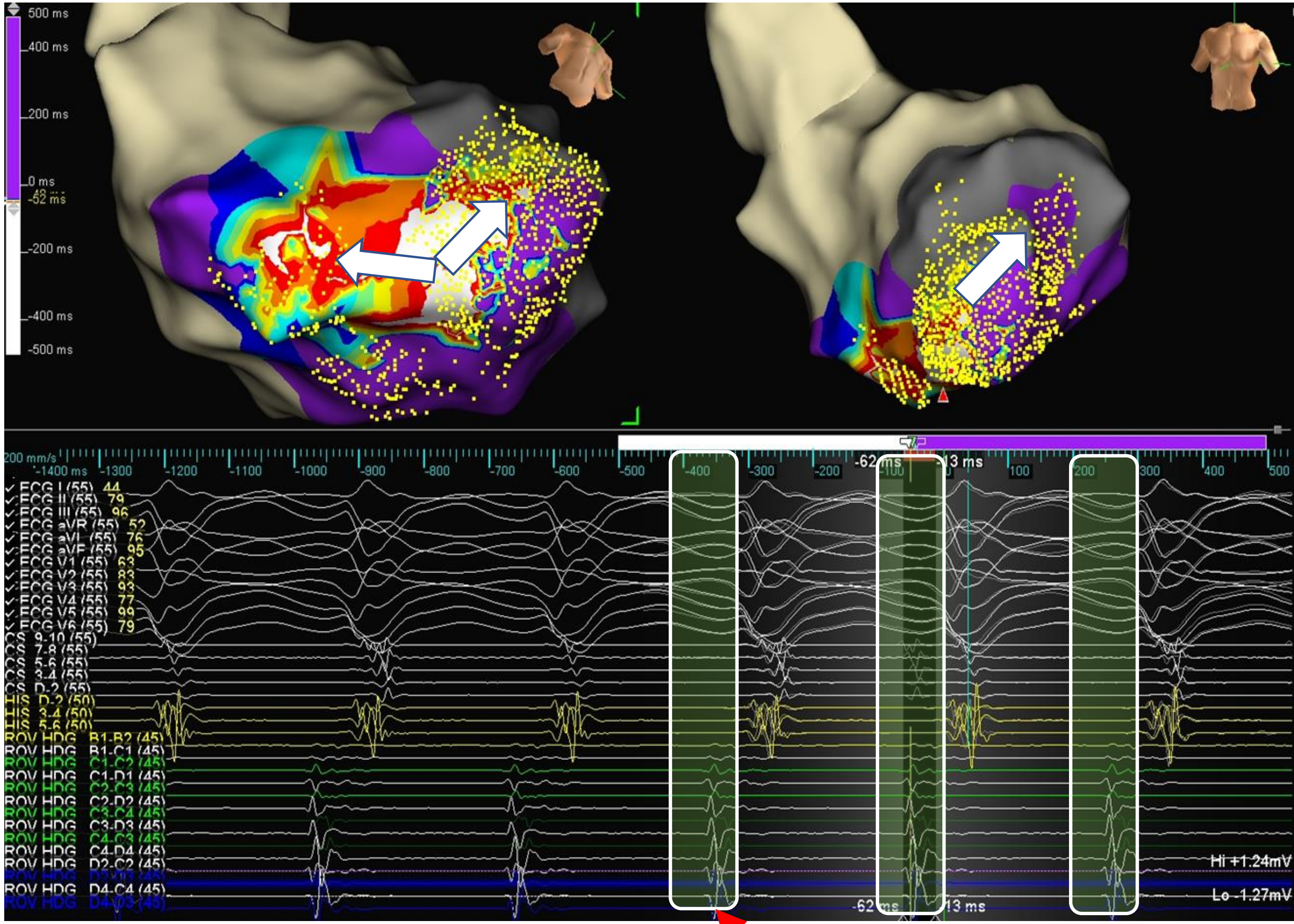

Pre-systolic phase

Supplementary figure 4

A

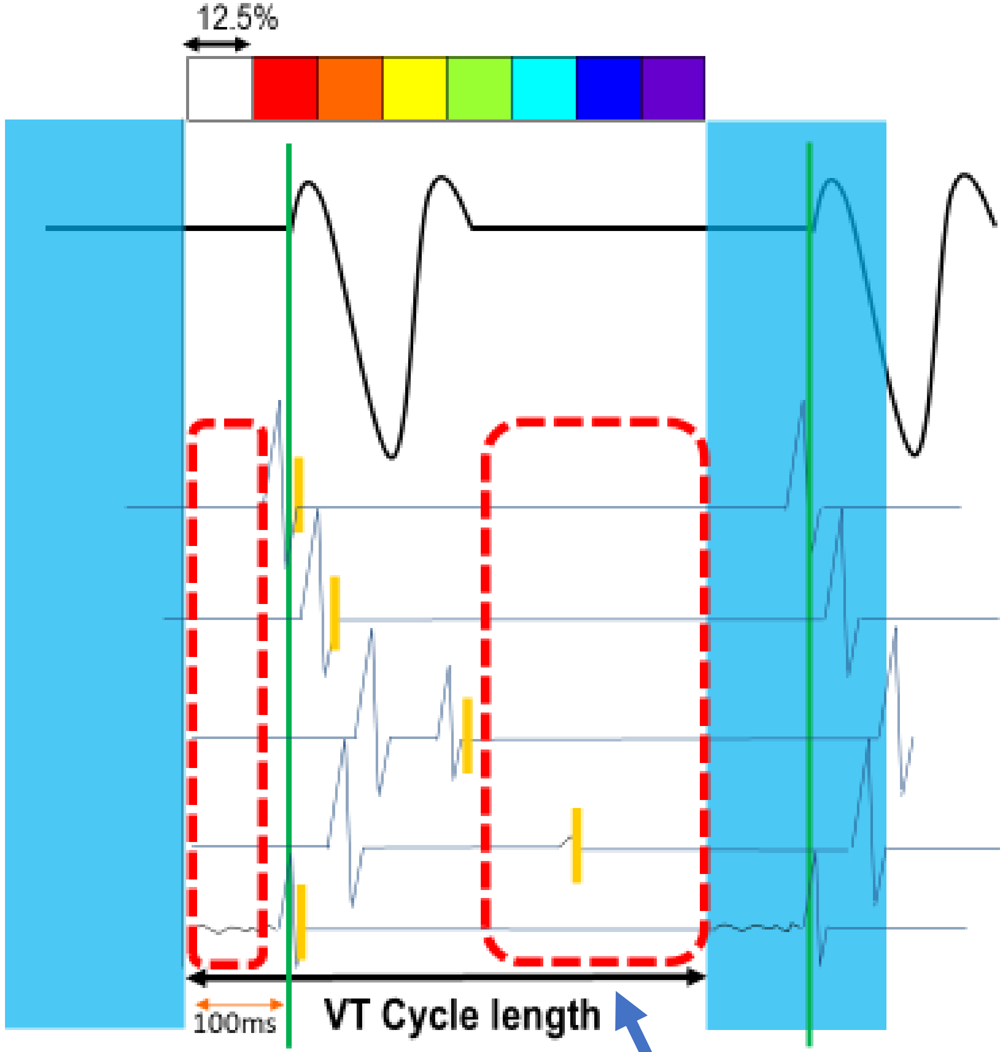

B

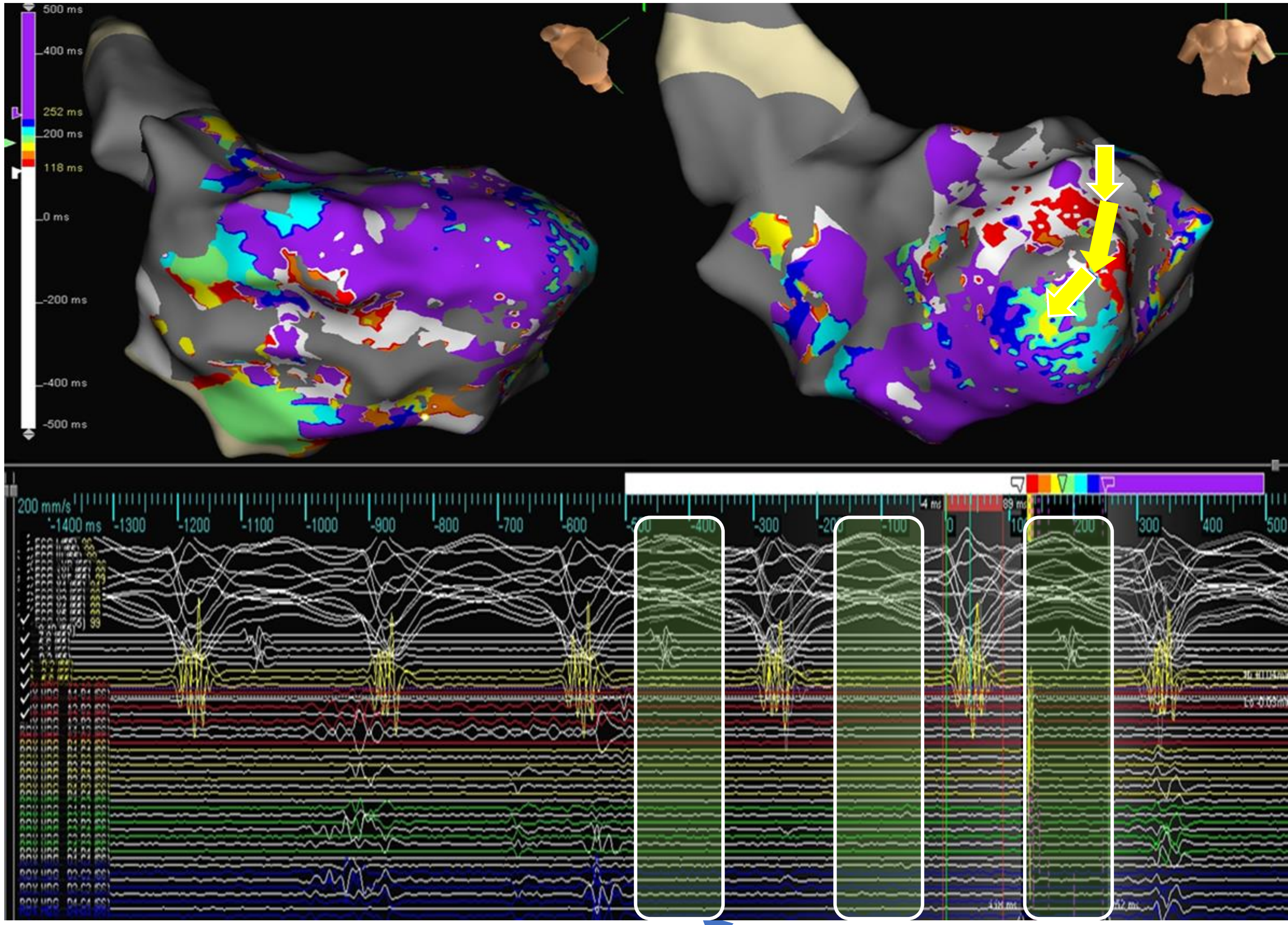

Mid-late diastolic phase

Mid-late diastolic phase

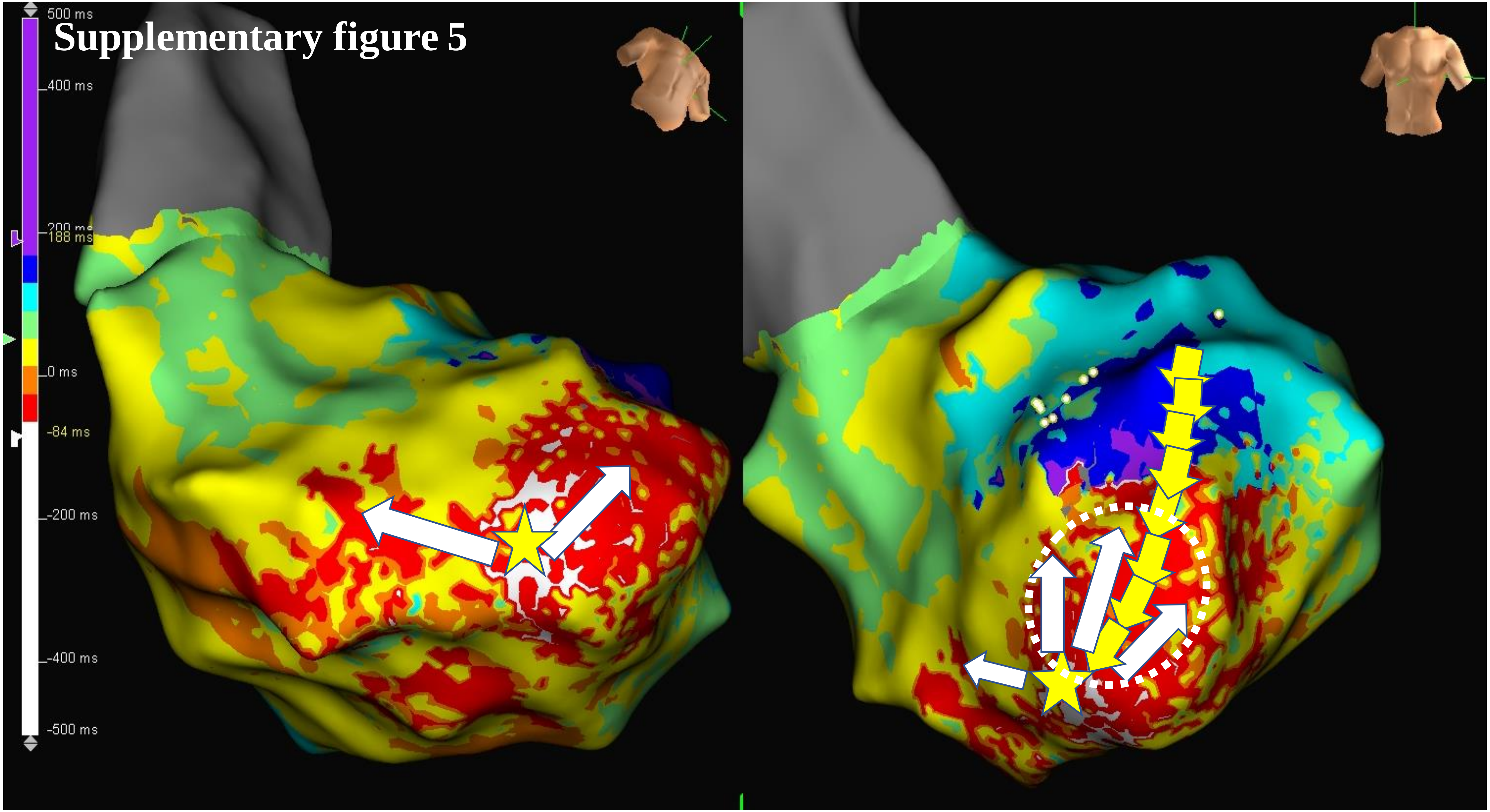

Pre-systolic potential   Mid-diastolic potential   VT exit
