# Supplementary material for: Use of a high-density mapping catheter for Purkinje-related ventricular tachycardia in a patient with a previous history of anterior myocardial infarction
Source: HeartRhythm Case Rep. 2021 Jan 20;7(4):232–6. doi: 10.1016/j.hrcr.2021.01.007 (PMC8129040; doi:10.1016/j.hrcr.2021.01.007)
Supplement: Supplementary Figure Legend [file mmc2.docx]

**Supplementary Figure legends**

**Supplementary Figure 1.**

A 3D image showed that the linear duodecapolar catheter was located on the scar area.

**Supplementary Figure 2.**

A fluoro image showed that the linear duodecapolar catheter was located in the left ventricle via the aortic valve.　The duodecapolar catheter was located in the left posterior fascicular region (A)(B), at the VT isthmus site (C)(D), and at the VT entrance site (E)(F).

**Supplementary Figure 3A**.

The interval window was set in accordance with the tachycardia cycle length (TCL) of the ventricular tachycardia (VT). This window (Pre-systolic phase) was set at a timing preceding the onset QRS by 100ms. (red arrow）

**Supplementary Figure 3B**.

During the pre-systolic phase, the impulse propagated retrogradely from the VT exit site to the left posterior Purkinje fibers (white arrow), and simultaneously another propagated from the VT exit site to the apical-anterior wall site via the endocardium.

**Supplementary Figure 4A**.

The interval window was set in accordance with the tachycardia cycle length (TCL) of the ventricular tachycardia (VT). This window (Mid-late diastolic phase) was set at a timing from the QRS offset to the timing of the initial pre-systolic phase. (blue arrow）

**Supplementary Figure 4B**.

During the mid-late diastolic phase, the impulse propagated from the anterior wall to the septal wall site (yellow arrow).

**Supplementary Figure 5.**

The activation pattern of the ventricular tachycardia (VT). The impulse propagated in the opposite direction. The propagation patterns of the pre-systolic potential and mid-late diastolic potential are demonstrated with white and yellow arrow respectively.

**Supplementary Video File**.

Activation pattern of ventricular tachycardia (VT). The activation map revealed that the impulse propagated retrogradely from the VT exit site to the left posterior Purkinje fibers and another propagated from the VT exit site to the apical-anterior wall site via the endocardium. The impulse that propagated retrogradely via the Purkinje fiber entered from the endocardial side of the apical-anterior wall region surrounded by the scar regions, then propagated on the epicardium to the septum, and finally exited from a distal site of the left posterior fascicule area.
